# Effect of high parity on occurrence of anemia in pregnancy: a cohort study

**DOI:** 10.1186/1471-2393-11-7

**Published:** 2011-01-20

**Authors:** Yahya M Al-Farsi, Daniel R Brooks, Martha M Werler, Howard J Cabral, Mohammed A Al-Shafei, Henk C Wallenburg

**Affiliations:** 1 Department of Family Medicine and Public Health, College of Medicine and Health Sciences, Sultan Qaboos University, Oman; 2 Department of Epidemiology, School of Public Health, Boston University, Boston, USA; 3 Department of Obstetrics and Gynecology, College of Medicine and Health Sciences, Sultan Qaboos University, Oman

## Abstract

**Background:**

Studies that explore the controversial association between parity and anaemia-in-pregnancy (AIP) were often hampered by not distinguishing incident cases caused by pregnancy from prevalent cases complicated by pregnancy. The authors' aim in conducting this study was to overcome this methodological concern.

**Methods:**

A retrospective cohort study was conducted in Oman on 1939 pregnancies among 479 parous female participants with available pregnancy records in a community trial. We collected information from participants, the community trial, and health records of each pregnancy. Throughout the follow-up period, we enumerated 684 AIP cases of which 289 (42.2%) were incident cases. High parity (HP, ≥ 5 pregnancies) accounted for 48.7% of total pregnancies. Two sets of regression analyses were conducted: the first restricted to incident cases only, and the second inclusive of all cases. The relation with parity as a dichotomy and as multiple categories was examined for each set; multi-level logistic regression (MLLR) was employed to produce adjusted models.

**Results:**

In the fully adjusted MLLR models that were restricted to incident cases, women with HP pregnancies had a higher risk of AIP compared to those who had had fewer pregnancies (Risk Ratio, RR = 2.92; 95% CI 2.02, 4.59); the AIP risk increased in a dose-response fashion over multiple categories of parity. In the fully adjusted MLLR models that included all cases, the association disappeared (RR = 1.11; 95% CI 0.91, 1.18) and the dose-response pattern flattened.

**Conclusions:**

This study shows the importance of specifying which cases of AIP are incident and provides supportive evidence for a causal relation between parity and occurrence of incidental AIP.

## Background

Despite being a major public health issue, anaemia in pregnancy (AIP) is, surprisingly, still not well understood in terms of its definition, prevalence, incidence, causes, and the effectiveness of iron supplements in improving pregnancy outcomes [1].

The uncertainty in defining AIP is a major obstacle in etiological research of AIP. Researchers show a tendency to confuse cases of anaemia caused by pregnancy (incident cases) with pre-existing cases of anaemia complicated by pregnancy (prevalent cases). This ambiguity stems from inconsistent systems of measurement criteria for the onset of anaemia. Some authors considered "*anaemia at first antenatal care visit*" as a measure of occurrence of AIP [2], while others considered *any antenatal *low hemoglobin (Hb) measurement throughout the course of pregnancy [3]. A third subset of reports provide no indication of which Hb measurement cutoff was used or the specific timing of onset measurement [4-6].

Ideally, a measure of incident cases of AIP should specify a point/period in time that is more recent than the onset of pregnancy and allows for a reasonable latency period for the causal action of pregnancy in causing incident AIP. To our knowledge, there is no universal standard for the exact timing of measurement for the onset of anaemia that would clearly differentiate between incident and prevalent cases.

Due to this variation in the definitions of AIP, estimates of the prevalence and incidence of AIP among pregnant women are uncertain. The World Health Organization (WHO) estimates the prevalence of anaemia among pregnant women to vary between 53.8% and 90.2% in developing countries, while in developed countries it is estimated to be 8.3% [7]. However, many of these women were already anaemic before being pregnant. As a matter of fact, the WHO estimates the prevalence of anaemia to be 47.5% among non-pregnant women in developing countries and 19% in women in developed countries [1,7]. Furthermore, the estimated prevalence of anaemia varies throughout the course of pregnancy. In the USA, for example, the prevalence of anemia among pregnant women is estimated to be 1.8% in the first trimester, 8.2% in the second trimester, and 27.4% in the third trimester [8].

Another factor that adds to the complexity in measuring the incidence of AIP is the variation among researchers in specifying the cutoff point of Hb level. While some investigators defined AIP as Hb < 11.0 g/dl as per the recommendation of the WHO [9,10], others adopted different cutoff points such as < 10.0 and < 10.5 g/dl which had been recommended by other parties in the USA [11,12].

High parity is among the factors with etiologic potential in causing AIP [13]. The WHO defines high parity (HP) as five or more pregnancies with gestation periods of ≥ 20 weeks, and low parity (LP) as less than 5 pregnancies with gestation periods of ≥ 20 weeks [14].

Prior studies provided inconsistent evidence regarding the question of whether high parity is associated with AIP. While some studies found that increasing parity was associated with an increase in the risk of AIP [5,6,15,16], others reported no evidence of such an association [4,17-19]. A third group of studies reported a reduction in risk of AIP [20,21].

This retrospective cohort study was conducted in order to explore the potential relation between parity and AIP with special attention to the distinction between prevalent and incident cases. The population studied were Omani woman characterized by a high prevalence of both HP and AIP.

## Methods

The study took place in Bidbid, a city located about 30 kilometres west of the capital, Muscat. This study was conducted in collaboration with an ongoing randomized community trial named: "Del**a**ying the Develop**m**ent of Di**a**betes Me**l**litus Type 2 in Oman", also called the "AMAL study". This project was launched in 2004, and it aims to estimate the prevalence of pre-diabetes among an Omani population and apply appropriate interventions to prevent the occurrence of diabetes. The AMAL study enrolled a total of 1313 subjects, 824 of whom were women. Among the female enrolees, 283 were nulliparous women and the remaining 541 were parous.

Our target was to enroll the 541 parous women and to collect relevant information about their pregnancies. Out of the 541 parous women, 532 (98.3%) agreed to participate after reviewing an informed consent. The study was approved by the Medical Research and Ethics Committee at Sultan Qaboos University.

The participants were asked to fill out a maternal health card (MHC) with details of all their pregnancies. These cards were our primary source of information for antenatal and clinical details pertaining to pregnancies. MHCs are registry cards that document all the events that occurred to the mother throughout pregnancy and after delivery. The cards contain the following sections: socio-demographics, pre-pregnancy risk factors, past medical history, obstetric & gynecological history, clinical findings at each visit, investigations, details of delivery, and post-natal findings.

The participants provided a list of all their pregnancies and the MHCs. After the exclusion of miscarriages, twin-pregnancies, and pregnancies < 20 weeks of gestation, the study's final population included 1939 singleton pregnancies with available MHCs among 479 women.

An incident case of AIP was defined as an episode of plasma hemoglobin level less than 11.0 g/dl first diagnosed in the second trimester or later, i.e. from 12 weeks onwards. The cutoff point was designated as 11.0 g/dl in accordance with the WHO recommendation and the local practice in Oman. The 12^th ^week was specified as the starting point of the eligible time frame for incident cases of AIP because it is during the beginning of the second trimester that pregnancy usually causes the steepest reduction in Hb level [22]. If a case of anaemia was diagnosed at booking or during the first trimester, it was thus considered to be a prevalent case for the purpose of this study and the pregnancy was excluded in order to limit the study population to those at risk of developing AIP.

Initial calculations of the cumulative incidence (risk) and the average hemoglobin level of occurrence of AIP were made for each level of parity, every single unit being treated as a level. The crude and adjusted measures of the effect of parity on the occurrence of AIP were obtained by using *multi-level *logistic regression (MLLR) analysis [23]. MLLR was preferred for analysis because it accounts for the dependency that exists among pregnancies that belong to the same woman.

The MLLR models were developed for AIP as an outcome using the hierarchical (PROC NLMIXED) regression modelling of SAS software (with a binomial distribution and logit link function). Two-level models were constructed which allowed for the grouping of pregnancy outcomes within women in order to include residuals for each pregnancy and for each woman. Thus the residual variance was partitioned into two components for each level, one showing the variance of residuals between different women and the second showing the variance of residuals between pregnancies in the same woman. This bi-level analysis revealed unobserved characteristics that affect pregnancy outcomes for the same woman, and it was these unobserved variables which showed the correlation between outcomes for pregnancies in the same woman. Variables deemed significant at p < 0.20 in a bivariate model were used in a multivariate model. A backward-selection procedure was then carried out, and variables meeting the p < 0.10 significance level were included in the final model. The odds ratios that were produced by the MLLR approximate the risk ratios which measure of effect of the relation between parity and AIP. In all MLLR models, goodness-of-fit was checked by via examining maximum likelihood estimates. The level of statistical significance was set at 0.05.

Two series of logistic regression models were conducted with different categorizations of parity. The first series treated parity as a dichotomous variable: LP (< 5) and HP (≥ 5). For the second series, parity was included as a categorical variable with the following categories: 1, 2-3, 4-5, 6-7, 8-9, and ≥ 10. With this categorization, we were able to evaluate if there was a dose-response relation between parity and risk of AIP.

Each series of analysis also included two sets of sub-analyses: a *crude *model and an *adjusted *model. In the crude model, parity was the only predictor of the occurrence of AIP. In the adjusted model, the following significant confounders were adjusted for: maternal age, maternal educational status, family income, past history of AIP, year of delivery, and inter-pregnancy time.

In order to explore the effect of changing the definition of AIP on the measures of effect of the relation between parity and AIP, two secondary analyses were conducted. First, the relationships were re-examined resetting the cutoff values for anaemia at hemoglobin levels < 10.5 g/dl and < 10.0 g/dl. Second, the study population was expanded to include all pregnancies with available MHCs (1939) and re-examined including these additional prevalent and incident cases of anaemia. All statistical analyses were performed using SAS software version 9.1 (SAS Foundation, Cary, NC).

## Results

The study population included 1939 pregnancies among 479 women. However, most of the analyses in this study excluded 591 of these pregnancies because they were associated with prevalent rather than incident cases of AIP. The study thus analysed the 1348 remaining pregnancies at risk of developing AIP among 341 women.

Table 1 compares important baseline characteristics of the included LP and HP pregnancies at the time of each pregnancy's occurrence. Out of the 1348 enrolled pregnancies, 38.7% were HP; these tended to be associated with higher maternal age. The majority of HP pregnancies occurred in women who were 25 to 35 years old, while the majority of LP pregnancies concerned women who were 20 to 25 years of age. Among HP pregnancies, 30.1% had a maternal age ≥ 35 years whereas among LP pregnancies only 2.6% of the women were ≥ 35 years of age.

**Table 1:** Comparison between LP (< 5) and HP (≥ 5) Pregnancies with available MHCs

	LP pregnancies		HP pregnancies	
Socio-demographic characteristic	Count	Percentage	Count	Percentage
n	826	61.3	522	38.7
Age				
15 to 24	504	61.0	58	11.1
25 to 29	237	28.7	132	25.3
30 to 34	64	7.7	175	33.5
35 to 44	21	2.5	157	30.1
Education				
Illiterate	158	19.1	304	58.2
Read only	152	18.4	101	19.3
6^th ^grade	171	20.7	59	11.3
9^th ^grade	172	20.8	39	7.5
12^th ^grade and higher	173	20.9	19	3.6
Monthly family income (Omani Rials)				
< 100	44	5.4	20	3.9
100 to < 200	330	39.9	123	23.6
200 to < 500	366	44.3	300	57.4
500 to < 1000	62	7.4	71	13.5
1000 and above	24	2.9	8	1.5
Year of delivery				
Before 1990	106	12.8	50	9.6
1990 to 1994	223	27.0	128	24.5
1995 to 1999	224	27.1	155	29.7
2000 to 2004	228	27.6	157	30.1
2005 and beyond	45	5.4	32	6.1
Past history of AIP	118	14.3	217	41.6
History of hematological disorders	53	6.4	61	11.7
Inter-pregnancy time (months)				
< 12	85	10.3	47	9.0
13 to 24	262	31.7	174	33.3
25 to 36	277	33.5	175	33.5
37 to 48	110	13.3	70	13.4
49 to 60	49	5.9	27	5.2
> 60	43	5.2	29	5.6

The rate of illiteracy among HP pregnancies was almost three times that of LP pregnancies (58.2% vs. 19.1%). While 62.5% of LP pregnancies occurred in women who attended standard schools, only 22.5% of the HP pregnancies occurred in women who attended standard schools.

HP pregnancies were associated with higher family income compared to LP pregnancies. The proportion of very low income (< 200 Omani rials) among LP pregnancies was 45.3% compared to 27.5% in HP pregnancies.

Among HP pregnancies, 41.6% had a positive past history of AIP which was three times that of LP pregnancies (14.3%). The proportion of pregnancies that had a positive history of hematological disorders in the HP group was almost twice that in the LP group (11.7% vs. 6.4%). Both LP and HP pregnancies tended to have a comparable distribution of inter-pregnancy time.

During the follow-up period, a total of 289 incident cases of AIP were enumerated among the 1348 pregnancies considered to have been at risk. Table 2 details the mean hemoglobin level and cumulative incidence (risk) of AIP for each category of parity. Overall, risk of AIP increases along with parity. The risk starts as high as 19.6 among the primiparous pregnancies. It then slightly drops over increasing parity units until parity 4, when the risk begins to increase steadily.

**Table 2:** Crude cumulative incidence (risk) of AIP over single units of parity

*Parity*	*hemoglobin level (g/dl) Mean (SD)*	*AIP cases*	*Pregnancies*	*Risk (per 100 pregnancies)*	*95% CI*
1	11.2 (0.9)	41	209	19.6	(14.7, 25.4)
2-3	11.4 (1.0)	44	441	10.0	(6.2, 13.9)
4-5	11.4 (0.9)	30	268	11.2	(9.1, 14.2)
6-7	11.5 (1.0)	57	190	30.0	(26.4, 33.7)
8-9	11.4 (1.0)	66	122	54.1	(49.6, 57.5)
10 and above	11.3 (1.0)	51	118	43.2	(40.1, 47.2)
**Total**	**11.4 (1.0)**	**289**	**1348**	**21.4**	**(19.3, 23.7)**

Table 3 shows the results of the first series of analysis, which treated parity as a dichotomy. The crude model showed that the risk of developing AIP among the HP pregnancies was more than four times higher than that among the LP pregnancies (RR = 4.37; 95% CI 3.32, 5.77). After adjustment for all confounders using MLLR, the risk of AIP among the HP pregnancies was still about three times higher (RR = 2.92; 95% CI 2.02, 4.59).

**Table 3:** Crude and adjusted logistic regression models for effect of parity on occurrence of AIP

	Crude		**Multi-level logistic regression**^**†**^	
**Parity**	**RR**	95% CI*	**RR**	**95% CI**

***Dichotomous parity***				
LP (< 5)	1.00	-	1.00	-
HP (≥ 5)	4.37	(3.32, 5.77)	2.92	(2.02, 4.59)
***Categorical parity***				
1	2.32	(1.45, 3.70)	1.09	(0.48, 1.88)
2-3	1.00	-	1.00	-
4-5	1.20	(0.73, 1.97)	1.14	(0.42, 1.99)
6-7	4.07	(2.61, 6.35)	3.01	(2.06, 7.02)
8-9	9.98	(6.95, 12.05)	5.67	(3.55, 13.16)
≥ 10	7.75	(4.78, 12.55)	4.32	(2.71, 14.25)

* RR refers to Risk Ratio; 95% CI refers to 95% confidence intervals.

† Adjusted for maternal age, educational level, family income level, year of delivery, past history of AIP, inter-pregnancy time, and past history of hematological disorders.

Table 3 also shows the results obtained by analyzing parity as a categorical variable. Using parity 2-3 as the reference category, the crude model showed that primiparity is associated with an increased risk of AIP (RR = 2.32; 95% CI 1.45, 3.70). As the level of parity increases, the risk ratios indicate a progressive increase in the risk of AIP. The highest risk of any parity category is observed among the parity 8-9 category (RR = 9.98; 95% CI 6.95, 12.05). The drop in the risk ratio observed in the parity ≥10 category is likely to be due to sparse data. Overall, the crude model strongly suggests a positive dose-response relation between parity and risk of occurrence of AIP.

Adjustment for confounders using MLLR showed a dose-response relation between parity and risk of AIP similar to that observed in the crude model, with the highest risk ratio again being observed in the parity 8-9 category (RR = 5.67; 95% CI 3.55, 13.16).

**Figure 1:**
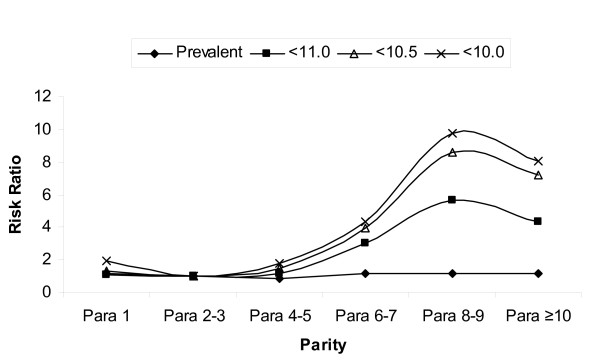
Results of adjusted analyses with varying definition of the outcome (AIP)

Table 4 and Figure 1 show the results of the secondary analyses with varying definitions of the outcome. Over dichotomous parity, the risk ratio increased slightly, from 2.92 for < 11.0 g/dl to 3.12 for < 10.0 g/dl, when the cutoffs were lowered. Over multiple categories of parity, taking < 10.5 g/dl as the cutoff value of the hemoglobin level produced a similar result pattern to that found with a cutoff value of 11.0 g/dl, although the RRs were higher. Taking < 10.0 g/dl as the cutoff value also produced a similar pattern with an even further increase in the RRs.

**Table 4:** Secondary adjusted analyses with varying definitions of the outcome (AIP)

	*All cases (prevalent & incident)*		*Incident cases only*					
		
			*Hb < 11.0 g/dl*		*Hb < 10.5 g/dl*		*Hb < 10.0 g/dl*	
**Parity**	**RR**^**†**^	95% CI*	**RR**^**†**^	**95% CI**	**RR**^**†**^	**95% CI**	**RR**^**†**^	**95% CI**

								
LP	1.00	-	1.00	-	1.00	-	1.00	-
HP	1.11	(0.91, 1.18)	2.92	(2.02, 4.59)	2.97	(1.93, 4.91)	3.12	(1.87, 5.25)
								
1	1.20	(0.92, 1.66)	1.09	(0.48, 1.88)	1.32	(0.63, 4.13)	1.97	(1.01, 12.03)
2-3	1.00	-	1.00	-	1.00	-	1.00	-
4-5	0.89	(0.55, 1.58)	1.14	(0.42, 1.99)	1.47	(0.41, 3.24)	1.76	(0.27, 5.78)
6-7	1.15	(0.58, 2.00)	3.01	(2.06, 7.02)	3.94	(1.52, 7.89)	4.35	(1.06, 14.57)
8-9	1.13	(0.30, 1.99)	5.67	(3.55, 13.16)	8.63	(2.70, 13.62)	9.73	(2.21, 29.61)
≥ 10	1.19	(0.41, 2.46)	4.32	(2.71, 14.25)	7.18	(2.54, 14.05)	8.05	(1.97, 26.04)

* 95% CI refers to 95% confidence intervals.

† RR obtained from MLLR in which we adjusted for maternal age, educational level, family monthly income level, year of delivery, past history of AIP, inter-pregnancy time, and past history of hematological disorders.

Table 4 also shows the effect of including prevalent cases of AIP in addition to incident cases. For the cutoff value of Hb < 11.0 g/dl cutoff value, adding prevalent cases attenuated the risk of AIP over dichotomous parity (RR shifted from 2.92 to 1.11). Over multiple categories of parity, the RRs either were unaffected or showed a slight increase in the risk of AIP over increasing parity units. The pattern suggested that including prevalent cases shifted the RRs towards null. See Figure 1 for comparison with results obtained from the primary analysis which was restricted to incident cases.

## Discussion

This retrospective population-based cohort study was conducted in order to explore whether parity has a harmful effect on the occurrence of AIP. Our results showed that HP pregnancies carry about three times higher risk of developing incident AIP than LP pregnancies, and that the risk of AIP increases in a dose-response fashion over increasing levels of parity.

The greater risk of AIP associated with higher parity may be explained by women having HP pregnancies' increased susceptibility to hemorrhage. In a healthy pregnancy, hormonal changes lead to an increase in plasma volume which causes reduction in hemoglobin level [22]. This hemodilution effect is considered normal if the hemoglobin concentration does not drop below a certain level e.g. 11.0 g/dl. Compared to the non-pregnant state, every pregnancy carries an increased risk of hemorrhage before, during, and after delivery. Therefore, higher parity exposes women more frequently to periods of hemorrhage risk. Although there is no consensus with regard to the exact mechanisms by which HP increases the risk of hemorrhage, some reports have suggested intermediaries such as increased venous drainage to the lower part of the uterus, hyalinization of blood vessels, and decreased elasticity of the uterine wall [24]. None of these proposed mechanisms have been confirmed [14].

Primiparous pregnancies were found to be at a higher risk of AIP compared to pregnancies with parity of 1 to 2. High rates of AIP among primiparous pregnancies are commonly found to be associated with adolescence and smoking [25]. Our finding could be explained by being adolescent as the majority (71.2%) were associated with a maternal of less than 20 years. Other risk factors such as active or passive smoking are unlikely to play a role since none of the women in our study reported active smoking, and the prevalence of smoking in our source population was 6.3%.

The study's results showed that the association between HP and AIP became more pronounced when the specificity of the definition of AIP was increased by lowering the diagnostic cutoff value. This finding emphasizes the importance of specifying an appropriate diagnostic cutoff value for AIP. Because one of the objectives of the antenatal care services is early detection of anemia in the course of pregnancy, some authorities, such as the WHO, recommend a high cutoff value in order to increase the sensitivity of the screening tests and thereby increase their ability to discover undiagnosed cases of AIP. Despite the utility of this recommendation in clinical practice, it is counter-productive for etiologic research. Adopting high diagnostic cutoff values reduces the specificity of the diagnostic test and increases the rate of false positives in the study, resulting in misclassification of the outcome via a form of information bias. In the present study, the results obtained with high cutoff values were shifted towards a null risk ratio because the misclassified outcome was non-differential over the categories of parity.

This study also found that including prevalent cases in the analysis tended to conceal the observed association between parity and AIP to disappear and flatten the dose-response curve. This finding may be explained by the fact that treating prevalent cases as incident cases reduces the specificity of the test, which again leads to a misclassified outcome. Since this misclassification occurred non-differentially over the LP and HP groups, the measures of effect were again shifted towards null [26].

Most of the previous studies did not adequately specify the timing and diagnostic cutoff value of AIP [3-6]. The main reason for this appears to be that those studies did not treat AIP as the main outcome, but rather listed it among many other antenatal complications. Their results thus provided inconsistent evidence of whether HP is associated with an increased risk of AIP.

While some studies provided information about their diagnostic criteria; however, significant methodological differences render any comparison of their findings with our results complex and generally unenlightening. Among these studies, the study conducted by Bugg et al. appears to have been the most comprehensive [2]: it was an age-matched retrospective cohort study in the United Kingdom conducted on a population of 794 women using two criteria to define of AIP cases: 1) booking Hb < 10.0 g/dl; and 2) any antenatal Hb < 10.0 g/dl. Beyond matching for age, there was no adjustment for confounders. With both definitions, an increased risk of AIP was found among HP women. The findings reported by Bugg et al. agree with our finding of an increased risk of AIP with HP; this agreement may be attributable to both studies' adoption of a highly specific definition of AIP.

The results of the present study are not without assumptions. By considering only pregnancies that had reached the 12^th ^week of gestation, we implicitly hypothesized a latency period of 12 weeks for the action of parity. This hypothesis might not be correct. Nonetheless, if the actual latency period is longer than 12 weeks, we would infer that our results were affected by non-differential misclassification of the outcome and were biased towards null. If this is the case, then actual measurements of the effect of parity would likely reveal an even higher risk of AIP among HP compared to LP pregnancies. On the other hand, the actual latency period is unlikely to be shorter than 12 weeks because the physiological mechanisms through which parity induces AIP, e.g. hemodilution and hemorrhage, have limited action before the second trimester [22].

Our results may have been affected by selection bias due to the exclusion of pregnancies with missing MHCs from the analysis. The possible existence and magnitude of such a selection bias was assessed by conducting a parallel analysis of pregnancies occurring only in women with no missing MHCs (data not shown). The results suggested a dose-response relation similar to that observed in the analysis of all pregnancies; it was therefore concluded that the impact of this selection bias, if any, was insubstantial.

## Conclusions

In conclusion, increasing parity appears to increase the risk of occurrence of AIP in a dose-response fashion. This study shows the importance of differentiating prevalent and incident cases of AIP. Inclusion of prevalent cases shifts the association towards the null and flattens the dose-response relation. Finally, the study shows the importance of clearly specifying a diagnostic cutoff value of hemoglobin level in defining AIP, as increased specificity of the definition enhanced the observed association.

## Competing interests

The authors declare that they have no competing interests.

## Authors' contributions

YMF formulated the study concept and collected data. He contributed to data analysis, literature review, and write-up of the manuscript. DRB and MMW conceptualized the methods and contributed in reviewing results and write-up of the manuscript. HJC conceptualized the regression modelling techniques, reviewed the results and contributed to the write-up. MAS contributed to the design and data collection in the field and contributed to the write-up. HCW revised the scientific background of the study and contributed to the literature review and write-up of manuscript, especially the Discussion. All authors read and approved the final manuscript.

## Pre-publication history

The pre-publication history for this paper can be accessed here:

http://www.biomedcentral.com/1471-2393/11/7/prepub
